# Neurotoxicity of organophosphate pesticides could reduce the ability of fish to escape predation under low doses of exposure

**DOI:** 10.1038/s41598-019-46804-6

**Published:** 2019-07-19

**Authors:** Natalia Sandoval-Herrera, Freylan Mena, Mario Espinoza, Adarli Romero

**Affiliations:** 1 Escuela de Biología, Universidad de Costa Rica, 11501-2060 San José, Costa Rica; 2 Central American Institute for Studies on Toxic Substances/Instituto Regional de Estudios en Sustancias Tóxicas (IRET), Universidad Nacional, Campus Omar Dengo, Heredia, Costa Rica; 3 Centro de Investigación en Ciencias del Mar y Limnología (CIMAR), Universidad de Costa Rica, 11501-2060 San José, Costa Rica

**Keywords:** Behavioural methods, Behavioural ecology, Conservation biology, Ecophysiology, Environmental impact

## Abstract

Biomarkers are frequently used in ecotoxicology as they allow to study toxicant effects happening at low concentrations of exposure. However, most sublethal studies only evaluate cellular biomarkers which lack evident ecological relevance. We used a multibiomarker approach to estimate the toxic effects of ethoprophos, an organophosphate insecticide commonly used in banana plantations, on the tropical fish *Astyanax aeneus* (Characidae). We measured biomarkers at sub-individual (cellular) and individual (metabolism, behavior) levels and examined relationships among these responses. A sublethal exposure to ethoprophos caused a significant (54%) reduction of brain Cholinesterase (ChE) activity, reflecting the pesticide’s high neurotoxicity. However, other biomarkers like oxidative stress, biotransformation reactions, and resting metabolic rate were not affected. Exposure to ethoprophos modified antipredator behaviors such as escape response and detection avoidance (light/dark preference): exposed fish escaped slower from a simulated attack and preferred brighter areas in a novel tank. The relationship between ChE activity and reaction time suggests that pesticide-induced ChE inhibition reduces escape ability in fish. Our results provide evidence that impacts of organophosphate pesticides on fish ecological fitness can occur even with short exposures at very low concentrations.

## Introduction

Tropical freshwater and estuarine systems are highly impacted by the continuous discharge of pesticides from agricultural activities, especially in regions with poor regulation and control like Central America^1–3^. In addition to low regulation, warm temperatures, high precipitation rates, and crop proximity to riparian environments make tropical ecosystems particularly vulnerable to pesticide runoff^4^. Organophosphates (OPs) is one of the groups of insecticides more widely used in agriculture. These compounds have high neurotoxicity on non-target organisms such as aquatic invertebrates and fish^5–8^. In fish, sublethal doses of OPs can cause physiological impairment of vital functions like feeding, predator avoidance, and reproduction^9^. Due to their short persistence (water DT50 = 20 days), OPs are usually found in low concentrations in aquatic systems. Therefore, to better understand the effects of OPs contaminants in an environmentally realistic context, ecotoxicity assessments should use low doses of exposure, similar to levels found in the field^10–12^.

A widely used tool to evaluate early, sublethal effects of pesticides exposure in fish, is the use of biomarkers^13–15^. Sub-individual biomarkers comprise subtle cellular processes related to the metabolism of xenobiotics (e.g., transferases, and hydrolases), as well as molecular alterations related to the mode of action of the substance (e.g., ChE inhibition, oxidative stress)^16^. Although these cellular biomarkers are often used to understand the mechanisms of toxicity, alone they are poor predictors of the ecological implications of pesticide exposure^17^. For this reason, it is necessary to evaluate simultaneously other responses at different levels of biological organization to understand how cellular disruption could affect systemic performance This integrative approach could help to better predict how sublethal toxicity at sub-individual and individual level could affect wild populations^15,18^.

The accurate selection of biomarkers is a key process to effectively link sub-individual responses to adverse individual outcomes. This choice should consider the toxicant’s mode of action, the physiological mechanisms and pathways potentially affected, as well as the specificity and sensitivity of the response to the toxicant^17,19^. Organophosphates, for example, have a very specific mode of action inhibiting Cholinesterase enzymes activity (ChE) in the nervous system, which makes it an ideal biomarker to detect exposure to this type of pesticides^11,13,20^. Cholinesterases are responsible for the hydrolysis of the neurotransmitter acetylcholine; in its absence, the neurotransmitter continues to stimulate the neuron causing and nervous overstimulation^21^. It is well known that the reduction of ChE activity disrupts various nervous processes, including muscle contraction, which could lead to disruption of swimming performance, muscle paralysis and respiratory failure in fish^21–24^. It is not known, however, whether exposure to environmentally relevant concentrations of OPs and the associated level of ChE inhibition, can still affect the individual performance of fish.

Aside from their neurotoxicity, organophosphates can increase cellular oxidative stress, by affecting antioxidant defense responses like catalase enzyme activity (CAT) and consequently leading to cellular damage e.g. membrane lipid peroxidation (LPO)^22,23^. Similarly, detoxification enzymes activity provide useful information about the metabolic pathways trigger by the toxicant. Glutathione S-transferases (GSTs), for example, catalyze the conjugation of xenobiotics with water-soluble substrates facilitating their removal^24^. The inclusion of diverse biochemical biomarkers improves the assessment of the OPs effects at the sub-individual level and potential mechanisms of their toxicity.

Most of these cellular alterations will affect systemic physiological processes at the individual level such as respiration, locomotion, and reproduction^25–27^. Resting Metabolic Rate (RMR) is a suitable biomarker to monitor physiological performance and energy expenditure under pesticide exposure conditions. Metabolic rate is a valuable indicator since energy trade-offs associated with pesticide removal and cellular repairing might reduce individual fitness^28–30^. In a more direct manner, assessing behaviour alterations allows to evaluate individual performance in an ecological context, providing a linkage between physiological processes and interaction with the environment^31–33^. Unsuitable behavioural responses can have severe implications for survival, providing more direct insights into the effects of pesticides at the population and community level^16,25,30^. Exposure to OPs, for example, has shown to affect swimming activity, feeding behavior, and predator avoidance in fish^27,34,35^.

Considering the usefulness of linking biochemical, physiological and behavioural endpoints to better predict the effects of toxicants exposure on fish populations, we proposed to use a suite of biomarkers and individual responses to study the toxic effects of ethoprophos, an organophosphate pesticide commonly used in banana plantations, on *Astyanax aeneus* (Characidae), a native freshwater fish from Central America. We examined ChE activity in brain and muscle, detoxification (GST) and oxidative stress (CAT, LPO) in liver, Resting Metabolic Rate and predator avoidance behavior. We also evaluated the relationship between cellular biomarkers and whole organism responses using statistical models that could help to identify relevant events for adverse outcome pathways.

## Results

### Biochemical biomarkers

**Figure 1:**
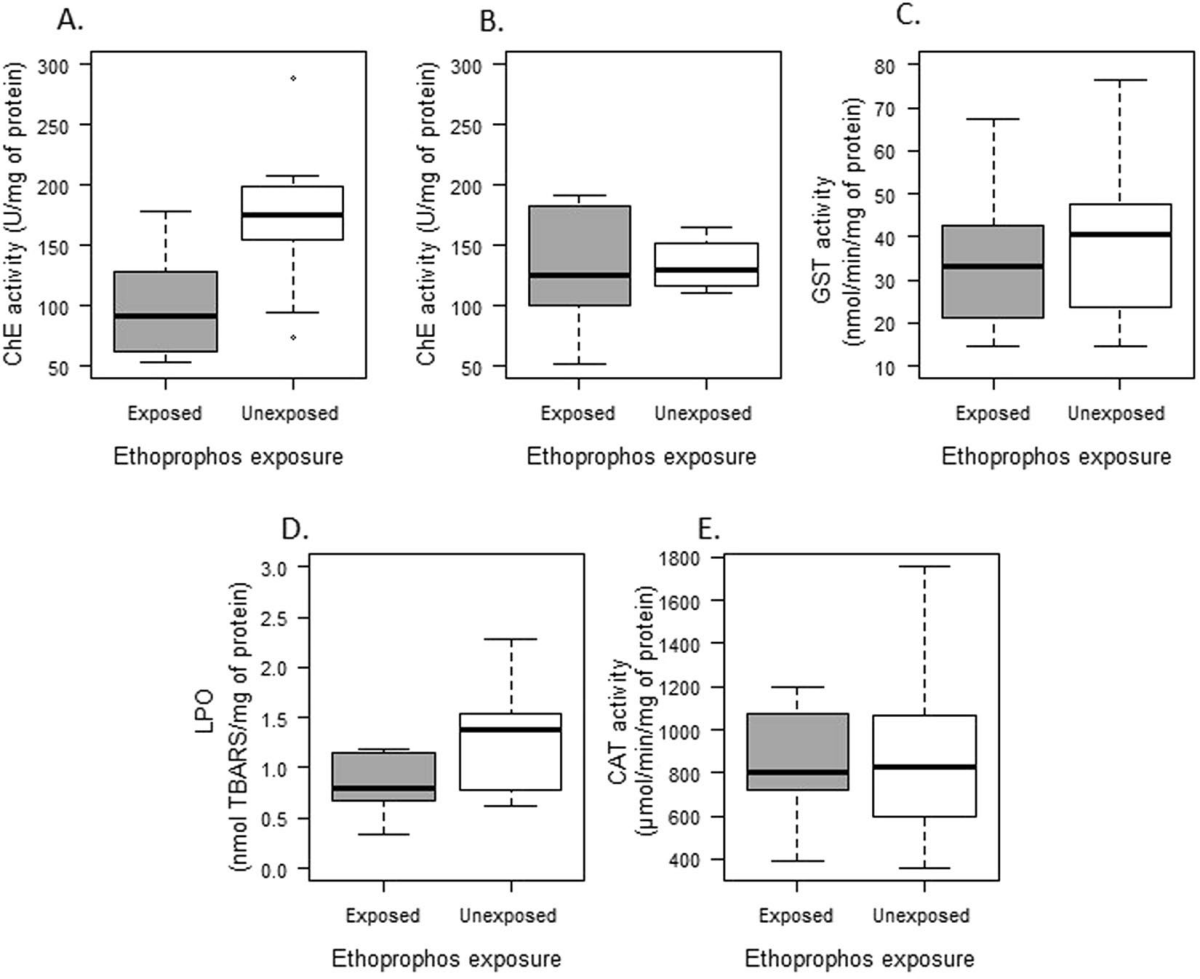
Biochemical biomarkers in *Astyanax aeneus* exposed and unexposed to a sublethal concentration of ethoprophos. (**A**) Cholinesterase (ChE) activity in brain. (**B**) Cholinesterase (ChE) activity in muscle. (**C**) Gluthatione S transferase activity in liver. (**D**) Lipid peroxidation (LPO) activity in liver. (**E**) Catalase (CAT) activity in liver. Error bars show the standard deviation of the mean.

Individuals exposed to a sublethal concentration of ethoprophos (0.01 mg.L^−1^ for 48 h) showed a significantly lower brain cholinesterase activity (ChE), 53,6% of inhibition, in comparison with unexposed fish (t = −3.390, df = 20, p = 0.003) (Fig. 1A). Conversely, muscle ChE activity was not significantly affected by ethoprophos exposure (t = −0.233, df = 18, p = 0.818) (Fig. 1B).

The acute exposure to ethoprophos did not have an effect on the induction of biotransformation enzymes, oxidative stress response nor antioxidant enzyme activity (Fig. 1C–E). No significant differences were detected between exposed and unexposed fish in GST (t = −0.606, df = 21, p = 0.5511), LPO (t = −2.091, df = 20, p = 0.059) or CAT (t = −0.159, df = 21, p = 0.874).

### Physiological biomarker

**Figure 2:**
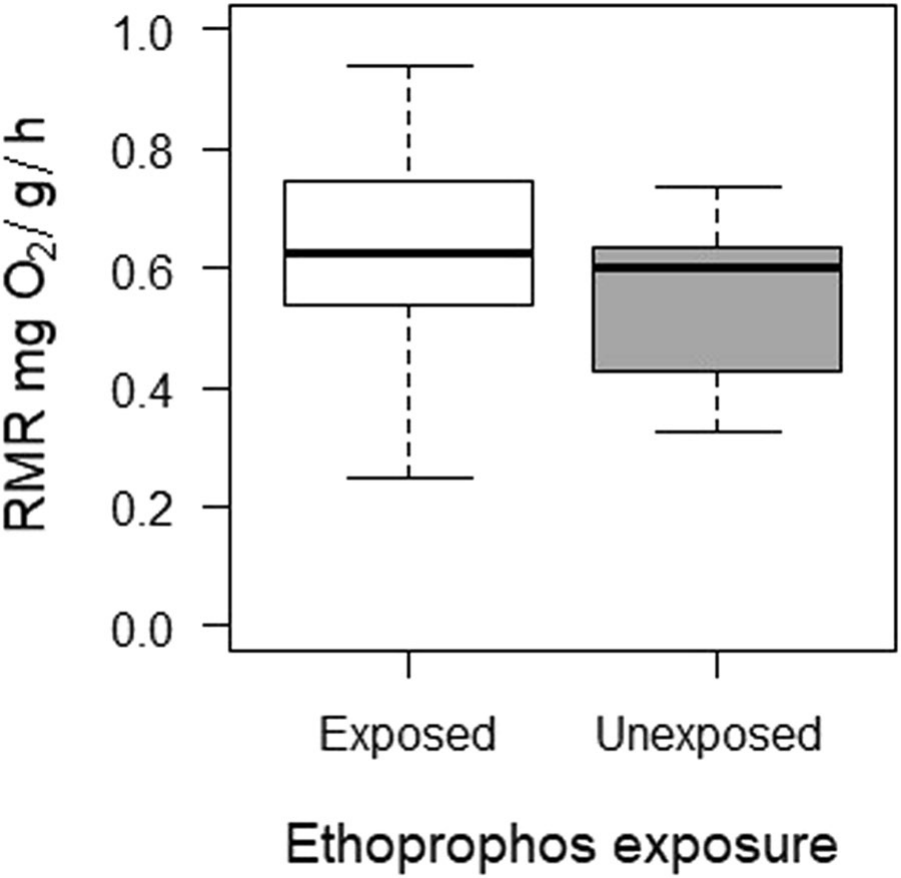
Resting metabolic rate of *Astyanax aeneus* exposed and unexposed to a sublethal concentration of ethoprophos. Error bars show the standard deviation of the mean.

Pesticide exposure did not affect fish metabolic rate. Similar resting metabolic rates (RMR) were found between exposed and unexposed fish (t = −0.617; gl = 21; P = 0.544) (Fig. 2). The RMR in *A*. *aeneus* was influenced by liver GSTs activity; individuals with higher GST activity exhibited lower metabolic rates (Table 1).

**Table 1:** General Least Square models explaining relationships between biochemical biomarkers and individual responses in fish exposed to a sublethal concentration of ethoprophos.

Effect	Value	SE	t-value	*P*
**Resting metabolic rate (RMR)**				
Intercept	0.849	0.065	13.152	<0,001
GST	−0.006	0.002	−4.148	<0,001*
**Detection avoidance behavior: time spent in light compartment (s)**				
Intercept	−2682.309	1526.684	−1.757	0.096
TL	498.931	260.991	1.912	0.072
ChE_M_	18.941	9.081	2.086	0.052
CAT	0.157	0.087	1.817	0.086
ChE_M_*TL	−3.388	1.555	−2.179	0.043*
**Reaction time (ms)**				
Intercept	9.693	1.568	6.178	<0.001
ChE_B_	−0.034	0.010	−3.326	0.003*
ChE_M_	0.013	0.011	1.196	0.245

Response variables: Resting metabolic rate (RMR), Detection avoidance behavior and Reaction time. Predictors: Gluthatione S transferase activity (GST). Total Length(TL). Muscle cholinesterase activity (ChE_M_), Brain cholinesterase activity (ChE_B_) and Catalase activity (CAT).

### Behavioural biomarkers

**Figure 3:**
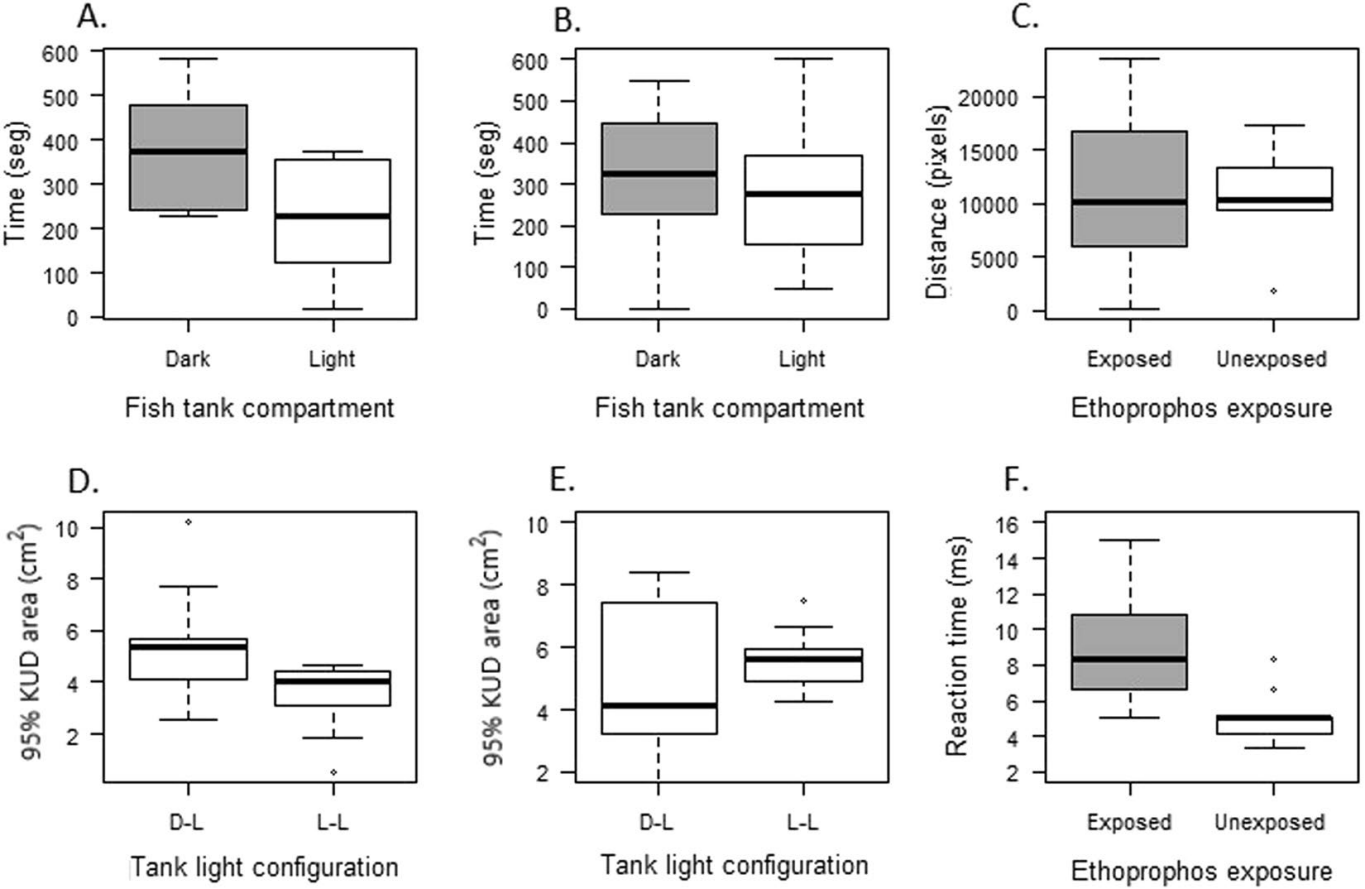
Behavioral biomarkers response of *Astyanax aeneus* exposed and unexposed to a sublethal concentration of the pesticide ethoprophos. (**A**) Time spent by unexposed individuals in the dark and light compartment during the detection avoidance behavior test. (**B**) Time spent by exposed individuals in the dark and light compartment during the detection avoidance behavior test. (**C**) Total distance traveled by exposed and unexposed fish during the test. (**D**) Total 95% Kernel utilization distribution (KUD) area used for exposed fish in each of the test configurations. (**E**) Total 95% (KUD) area used for unexposed fish in each of the test configuration. (**F**) Reaction time of exposed and unexposed individuals in response to a simulated attack of a predator dummy. Error bars show the standard deviation of the mean.

Detection avoidance behavior was affected by the acute exposure to ethoprophos. Unexposed fish avoided the light compartment of the tank, as expected (t = −2.4894; df = 20; p = 0.02172) (Light = 226.16 ± 136.96 s; Dark = 371.67 ± 137.23 s) (Fig. 3A). In contrast, exposed fish did not show a preference for one of the compartments, they explored both areas of the tank for similar time (t = −1.006; df = 22; p = 0.326) (Fig. 3B). A significant relationship between the time spent in the light compartment, as an indicator of the risk evaluation ability, and the interaction between muscle ChE activity and total length was found.

Fish locomotion, defined as the total distance traveled by an individual during the test, was not affected by the exposure to the pesticide (t = 0.17733, df = 20, p = 0.861) (Fig. 3C). Likewise, both exposed (V = 19, df = 10, p = 0.1294) and unexposed (V = 0.4300, df = 10, p = 0.6763) fish explored a similar size of the total area of the tank (95% KUD-kernel utilization distribution) during the light-dark test configuration (Fig. 3D,E).

The reaction time to escape from the simulated attack was significantly different between treatments (W = 16; p = 0.00174) (Fig. 3F). As expected, individuals exposed to ethoprophos responded slower (8.89 ± 3.43 ms) than unexposed fish (5.00 ± 1.49 ms). The reaction time of *A*. *aeneus* was not influenced by size (F_1, 21_ = 0.0819; p = 0.7775). However, there was a significant effect of brain ChE activity on the reaction time. Fish with reduction in the brain ChE responded slower to the dummy predator attack (Table 1).

## Discussion

To our knowledge, the present study provides the first ecotoxicological assessment for an organophosphate pesticide using a multibiomarker approach in a native tropical fish species. Our results indicated that a low acute sublethal exposure to ethoprophos in *A*. *aeneus* has significant effects on the fish physiology and behavior. These alterations in cellular and physiological responses varied in magnitude and occurrence, and in some cases suggested a cause-effect relationship among biomarkers.

### Neurotoxicity

As was expected Brain ChE activity was significantly reduced in exposed fish. This effect has been already reported for teleost species exposed to organophosphate pesticides, including ethoprophos^7,8,36–39^. The exposure dose we used was fifty times lower than the LC_50_ reported for this species, on organisms of similar age and collected in the same region^37^; even so, we found more than 50% inhibition of brain ChE_B_ activity, which demonstrates that harmful effects related to the mode of action of the substance can be triggered at a small fraction of lethal concentration.

Despite the inhibition observed in brain ChE activity, muscle ChE activity was not significantly affected by the ethoprophos exposure. Our results are in concordance with the effect observed in *Astyanax* sp. exposed to other organophosphate herbicides^40^. Since cholinesterase enzymes are polymorphic, brain and muscle isoforms would have different sensitivity toward anticholinesterase agents such as organophosphates^20,41^. Brain AChE, the enzyme that breaks down ChE, has been found to have a higher sensitivity to these inhibitors in comparison to the muscle isoform, which supports our findings^42^.

### Biotransformation, oxidative stress, and antioxidant enzymes

An increase in GST activity is used as a defense biomarker of pollutant exposure and oxidative stress^43^ Even though some authors have reported an increase in GST activity in fish exposed to organophosphorus pesticides^7,44,45^, we did not find a difference in the GST activity in fish exposed to ethoprophos. This result might indicate that the GSTs biotransformation pathway is not induced by ethoprophos, at least at this sublethal concentration and with short exposure time (48 h)^40^. On the other hand, GSTs are a multigene protein family with high polymorphism occurrence, which may influence the enzyme’s sensitivity to xenobiotics. This fact might allow some individuals to deal more efficiently with pollution exposure, impeding to detect significant changes in the overall GST activity^46,47^.

Oxidative stress occurs when the rate of reactive oxygen species (ROS) generation exceeds the antioxidant defense system^23^. Fish exposed to ethoprophos did not show significant changes in lipid peroxidation, indicating either that the exposure did not increase ROS production or that the antioxidant protective response was efficient enough to counteract oxidative stress^12,44^. To determine if this antioxidant response was induced we quantified the activity of the enzyme catalase CAT, but no differences were found between exposed and unexposed fish either. This result may support the idea that the ethoprophos exposure did not increase the production of ROS. However, specific quantification of ROS and a wide variety of oxidative stress biomarkers would be required in order to test this hypothesis.

### Metabolism

Coping with chemical exposure involves cellular processes that could require a high energy investment^25^. However, energy expenditure and allocation are highly regulated by homeostatic mechanisms, since energy balance determines individual fitness^45^. Significant changes in energy balance could only be evident when the homeostatic mechanisms fail, for example, changes in behavior like a reduction of swimming speed and decrease of respiration rate^48^. Although some studies have demonstrated changes in metabolic rate after exposure to organophosphates^49–52^, in our study, a very low concentration of ethoprophos did not affect the RMR in *A*. *aeneus*. The short time of exposure and the frequency of the dose might not be stressful enough to generate an energy imbalance detectable in the resting metabolic rate measurement. The fact that ethoprophos did not induce biotransformation and antioxidant proteins could support this hypothesis. Future studies should examine the effects of multiple subsequent acute exposures on fish metabolic rates.

It is important to consider that differences in metabolic rate among treatments might be difficult to detect due to the high intraspecific variation attributed to genetic variation, maternal effects, and epigenetic regulation^25,53,54^. Behavioural differences among individuals may also affect estimations of RMR during respirometry. For example, some individuals are more ‘reactive’ than others when confined in respirometers, which may result in an overestimation of the RMR^55^. Since any treatment effects on RMR are hard to distinguish from intrinsic variability, this biomarker might not be suitable for studying low-acute dose exposure effects.

#### Detection avoidance behavior

The light/dark test is based on the natural preference for dark environments in detriment of bright ones, as has been documented in some fish species^56–58^. This behavior is known as scototaxis, and it works as a defense strategy to avoid predators since dark places make it harder to distinguish them from the substrate^56,58,59^. Unexposed fish showed aversion for the light side of the tank, supporting scototaxis behavior previously reported for other species of *Astyanax*^57^. *Astyanax aeneus* usually hunts insects in shallow and surface waters, where they can suffer predation from birds. Therefore, dark sites may represent protected environments, which may favor scototaxis in this species. Likewise, the uniform pattern of swimming exhibited in both sides of the tank might reflect an approach-avoidance conflict between its natural tendency to explore a new environment, where food and sexual partners might be found, and its instinct to seek shelter^56,59^.

Scototaxis has also been used to study anxiety in fish. In general, anxiolytic drugs and treatments increase the time the animal spends in the white compartment, while anxiogenic drugs decrease this time^60,61^. We also used these parameters to estimate the effect of ethoprophos in scototaxis and locomotion in *A*. *aeneus*. In contrast to unexposed fish, exposed fish did not show a preference for one of the compartments in the D/L box test, suggesting an impairment of scototaxis behavior by the ethoprophos exposure. Based on the anxiety evaluation premise, ethoprophos diminishes anxiety in fish, allowing the fish to explore the unprotected compartment of the tank for longer. Most research has studied GABA and serotonin pathways as pharmacological mechanisms controlling anxiety in fish. However, there is growing evidence that the cholinergic system may also play a modulating role in anxiety^62,63^. We suggest that the accumulation of Acetylcholine in the CNS, caused by ChE inhibition, might function as an anxiolytic agent in exposed fish and could explain the behavioral changes observed. This modification of the environment selection in *A*. *aeneus* could increase the risk of predation in polluted environments.

#### Antipredator behavior

Predator-prey interactions are fundamental in the dynamics of ecosystems. These trophic relationships define behavioral patterns of organisms^64–66^ and when an environmental stressor disrupts such patterns, it can have ecological implications. Organic pesticides and particularly organophosphates have been shown to affect the ability of fish and aquatic invertebrates to avoid predation. In our study, fish exposed to ethoprophos had a slower escape response to the simulated predator attack than unexposed fish. In natural conditions this delay to elude the attack would make them more susceptible to predation, potentially increasing mortality, which could eventually affect the population size and structure^67^.

The escape response in most fish is known as C-startle reflex. This avoidance behavior is mediated by Mauthner cells (M-cells), command neurons that receive acoustic/vibrational stimuli and in response elicit a contralateral muscle contraction; this allows the fish to quickly C-bend and bout, escaping from the potential dangerous stimulus^68^. Since the synapses leading the muscle contraction are cholinergic, the C-startle reflex is susceptible to ChE inhibitors^69,70^. In fact, OPs like Chlorpyriphos can affect the escape response in Medaca by impairing the communication between M-cells and the neuromuscular junction^71^. This mechanism of toxicity is consistent with the relationship we found between brain cholinesterase inhibition and reaction time in fish exposed to ethophrophos. The correlation between ChE activity, a biochemical response, and avoidance behavior as an individual endpoint, provides evidence that the detection of brain ChE inhibition could ultimately predict a decrease in organism survival.

#### Ecological significance

Using a multibiomarker approach should facilitate the elucidation of possible cellular or physiological mechanisms of toxicity underlying the effects observed at higher levels of biological organization. We found a significant correlation between the effect of ethoprophos at a biochemical level (inhibition of brain ChE activity) and a behavioral response (antipredator behavior) that might contribute to explain how exposure to sub-lethal concentration of organophosphates can affect the ecological fitness of fish. This relationship has been reported in other species exposed to pesticides, however, this is the lowest concentration at which it has been observed^72–74^. Predator-prey interactions are among the main elements molding community structure and, therefore, can function as important links between toxicant-induced effects on individuals and effects at higher levels of organization^35^. Likewise, these interactions provide valuable information for biotransference and bioaccumulation dynamics in aquatic ecosystems^75^. For instance, individuals highly affected by pollutants exposure would be easier for predators to capture, which can facilitate the transfer of the contaminants to higher trophic levels^35^.

## Conclusions

Our results showed that exposure to a low sublethal concentration of the organophosphate pesticide ethoprophos has a neurotoxic effect on fish that correlates with a slow escape response to predator attack. While ChE activity has often been used as a biomarker of neurotoxicity in fish, it has rarely been correlated with effects at the organismal level. Therefore, this study supports the usefulness of ChE activity as a biomarker under environmental realistic exposures, especially when it is accompanied by organismal level biomarkers such as behavior. Likewise, we propose the reaction time test as a valuable tool for screening of chemical compounds potentially affecting predator avoidance behavior. Screening multiple biomarkers with an integrative approach, helps to improve our understanding of how toxicants affect different levels of biological organization and could be also used to assess the effects of other environmental stressors such as climate change on wildlife.

Currently, most regulations for the use of pesticides are based only on acute toxicity (e.g., LC50), however, our results give strong evidence that impairments on fish behaviour occur even at short exposures at very low concentrations. We propose that sublethal effects with potential ecological implications must be considered to develop effective policies for sustainable pesticide use and ecosystem health protection.

## Materials and Methods

### Study species and fish collection

*Astyanax aeneus* (Characidae) is a freshwater fish native from Costa Rica and widely distributed from southern Mexico to western Panama^76^. This species has been proposed as a good sentinel organism for ecotoxicology studies in the region due to its small size, high abundance and wide distribution^19,37^.

Thirty *A*. *aeneus* were caught in a freshwater lagoon located in Sarapiquí, Heredia (10.378705°N, 83.944738° W), using a fish trap with bait. Only mature individuals of similar size (>4 cm TL) were collected since body size can influence antipredator behavior^61^ and enzyme activity^77^. Fish were transported in plastic containers with continuous pump aeration to the Universidad Nacional (Heredia, Costa Rica). Temperature (°C), pH, dissolved oxygen (mg/L), and conductivity (μS/cm) were measured *in situ* using a portable multi-probe (Hach HQ40 D). Superficial water samples were also taken using pre-washed 1-L brown glass bottles. The bottles were transported in cooled ice boxes to the laboratory LAREP (Laboratorio análisis de residuos de plaguicidas), UNA, Heredia, Costa Rica and stored at 4–6 °C until analyses of pesticide residues.

### Experimental design

Fish were exposed to 0,014 mg/L of ethoprophos for 48hrs (see supplementary material for more details). After this period, they were transferred into experimental tanks where the metabolic and behavioral experiments took place. The sequence of the experiments was the following: (1) resting metabolic rate (RMR) measurement; (2) detection avoidance behavior assessment; (3) antipredator behavior trials; and (4) tissue extraction for cellular biomarkers quantification. All measurements were performed at the same time from 8:00 am to 11:00 am to avoid changes attributed to circadian activity patterns^45^. All the biomarkers were evaluated in 30 *A*. *aeneus* ranging in size from 4 to 6 cm TL. None of the individuals had a significant presence of ectoparasites or lesions in their body that could have interfered with their behavior and physiological response to the pesticide exposure^78^. Ethoprophos concentration was measured in the exposure tanks at the beginning and the end of the experiments (see supplementary material)

### Biomarkers

#### Biochemical biomarkers

Following the metabolism and behavior tests, we collected the following data for each fish: weight (g), total length (LT), standard length (LE), volume and sex. Individuals were then decapitated and brain (whole), muscle (≈50 mg from the base of the first dorsal fin) and liver (whole) samples were collected and stored at −80 °C until chemical analysis was conducted at the Ecotoxicology Laboratory of the National University (ECOTOX-UNA). Cholinesterase (ChE) activity was quantified in brain and muscle samples. Glutathione S-transferase (GST) activity, lipid peroxidation (LPO) and catalase activity (CAT) were measured in liver tissue.

Samples were homogenized in an appropriate buffer and processed as described in Mena *et al*.^37^ for biomarker determinations. Briefly: protein content in sample homogenates was determined by the method of Bradford^79^ a Sigma^®^ reagent and bovine serum albumin (BSA) as standard. ChE activity was measured using the method of Ellman *et al*.^80^, using 1 mM acetylthiocholine and 0.1 mM 5,5′ dithiobis-2-dinitrobenzoic acid (DTNB) as substrate and conjugate; the reaction was measured at 415 nm during 15 min and expressed as nmol/min/mg protein. GST activity was determined as described by Habig *et al*.^81^, exposing samples to 1 mM CDNB and 1 mM GSH and monitoring the reaction at 340 nm during 3 min.; activity reported as nmol/min/mg protein. Lipid peroxidation was measured by the thiobarbituric reactive species (TBARS) assay^82^ and expressed as nmol TBARS per mg of protein. CAT activity was measured according to Aebi *et al*.^77^ by the decrease in absorbance at 240 nm during 20 seconds due to H_2_O_2_ consumption and expressed as µmol/min/mg protein.

#### Physiological biomarker: resting metabolic rate (RMR)

**Figure 4:**
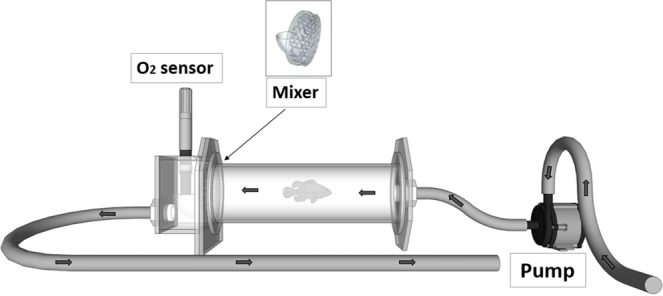
Diagram of the chamber used for the intermittent respirometry. The thick arrows show the water flow and the position of the mixing device. The arrow shows the position of the mixing device.

The resting metabolic rate (RMR) was measured using an intermittent flow respirometry (Fig. 4). This set-up consisted of a series of short-term, closed respirometer experiments, interrupted by flushing intervals that allow the water in the chamber to be replaced with oxygenated water. The closed periods lasted approximately 30 min (10% decrease in dissolved oxygen at 25 °C) while the water in the chamber was circulated in a closed loop without external water input. Water oxygen was measured every five minutes at constant temperature (25 ± 1 °C), using a dissolved oxygen probe (YSI 550 A-12, polyethylene membrane, measurement range 0 to 50 mg/L). The oxygen consumption was calculated from the slope of dissolved oxygen as a function of time. By using the slope more data is included in the calculation, which provides a better fit than just considering the oxygen at the beginning and at the end of the trial^83^.

The resting metabolic rate (RMR) was calculated using the following formula:

$$RMR(\frac{mg\,{O}_{2}}{g\,h})=(\frac{Slope(\frac{mg\,{O}_{2}}{L\,h})\,\ast \,{V}_{chamber}(L)}{Fish\,weight(g)})$$

The slope value corresponded to the linear decrease in oxygen content during the time the chamber was closed and *V*_*chamber*_(*L*) = *V*_*Flask*_(*L*) − *V*_*fish*_(*L*). The volume of the flask *V*_*Flask*_(*ml*) was the total volume (L) of the empty respirometer, including the recirculation loop. *V*_*Fish*_(*ml*) was the volume of water that is displaced when the fish is placed into the chamber. This volume was estimated by submerging the fish in a known volume.

### Behavioral tests

#### Detection avoidance behavior: dark-light box model

**Figure 5:**
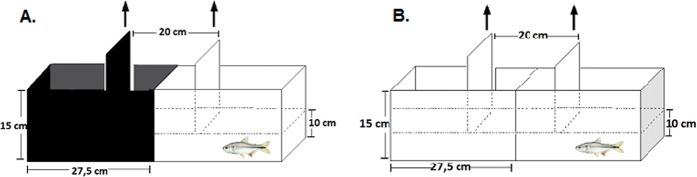
Setup for Light/dark box test used to evaluate the detection avoidance behavior in *A*. *aeneus*. (**A**) Fish tank design for the light/dark. (**B**) Fish tank design for the light/light

The dark/light preference protocol is a behavioural model for fish that is being validated to assess the behavioural effects of toxic substances, and it is based on fish natural preference for dark places^84^. The experimental setup consisted of an equally divided half-white, half-black 20 L tank (40 cm long × 25 cm high × 20 cm wide), named as light-dark configuration (Fig. 5A). Fish were placed in an intersection compartment, located between one white and one dark compartment. This intersection compartment is enclosed by two sliding doors, which were removed following a habituation interval of 5 min. The fish was then allowed to explore the tank freely. Exposed and unexposed fish behavior was recorded for 10 minutes using a webcam shooting 30 frames per second. An all-white experimental tank was used as negative control and was named as light-light configuration (Fig. 5B). The water used in the test was saturated with oxygen and de-chlorinated to avoid oxygen reduction during the test.

The behavioral footage was analyzed using a background subtraction and blob detection algorithms from the openCV library in Python^85^. Coordinates (X/Y) from individual fish were extracted in each frame of the video. The illumination preference was defined as the average time each individual spent in a specific compartment of the experimental tank. The fish locomotion was evaluated using two parameters: (1) the total distance traveled during the detection avoidance test, and (2) the 95% of the area used in the fish tank during the test (95% KUD - kernel utilization distribution). Preference and locomotion analyses were performed in the Mathematica software (Wolfram Mathematica, license U.C.R).

#### Antipredator behavior

**Figure 6:**
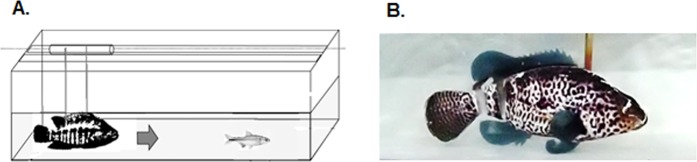
Antipredator test setup used to evaluate the escape behavior of *A*. *aeneus*. (**A**) Experimental tank design. (**B**) Predator dummy used to simulate natural predator *Parachromis managuensis*.

The antipredator behavior was evaluated measuring the fish escape response to a simulated predator attack. As a predator, we used a fabricated 3D printed model (dummy fish) of the spotted guapote (*Parachromis managuensis*), a well-known natural predator that inhabits the same environments as *A*. *aeneus* (Fig. 6). The stimulus applied to trigger the escape response was a vibration generated by the bow wave flow when the dummy fish suddenly approaches. The dummy was released automatically generating a disturbance in the water, thus simulating a sudden attack. The predator attack and fish response were recorded using a digital camera configured to record at 60 frames per second. Slow motion recordings allowed quantifying more accurately the moment the attack started and the subsequent escape response of *A*. *aeneus*. The reaction time of *A*. *aeneus* to the potential attack or interaction with the predator (*P*. *managuensis*) was defined as the time period between the start of the event and the escape signal (i.e. flexion of the caudal peduncle of *A*. *aeneus*)^86^.

### Statistical analysis

For each of the quantified biomarkers, comparisons between treatments (exposed, unexposed) were investigated using t-tests. Nonparametric Wilcoxon test was used when the normality and homoscedasticity assumptions were not fulfilled. For the detection avoidance behavior analysis, differences in the time and 95% KUD area in the light-dark and light-light tank configurations were evaluated with a Wilcoxon ranked test and paired Student’s t-test, following the criteria mentioned above. The Wilcoxon matched-pairs signed-rank test was used to determine the differences between the time spent in the black and white compartments. Statistical significance was evaluated at α = 0.05 level. The values are reported as a mean ± standard deviation. Values above or below two standard deviations were considered outliers and thus were excluded from the analysis. Statistical analyses were performed using the R statistical package (R Development Core Team 2015).

A Generalized Least Squared model (GLS) was conducted to investigate which of the biochemical biomarkers responses influence the physiological and behavioral responses. The RMR, the reaction time and the time spent in the light compartment were used as the dependent variables. Each of them was tested as function of the following predictors: body size (TL), ChE_B_, ChE_M_, GST, LPO, and CAT. These variables were added using a stepwise algorithm and the best model was selected using the AIC (Akaike’s) and BIC (Bayesian Information Criterion) criteria. Graphical analyses of data and residual plots were used to evaluate the final regression model compliance with statistical assumptions. These analyses were performed using the library nlme from the R statistical package (R Development Core Team 2008).

### Ethical approval

All the procedures in this study were approved and carried out in accordance with the laboratory animal care protocol from the Comité Institucional para el Cuidado y Uso de los Animales (CICUA). The fish capture was approved by local authorities of Costa Rica (SINAC-ACLAC-PIME-VS-R-015-2016).

## Supplementary information

Supplementary material

## Data Availability

The datasets generated and/or analyzed during the current study are available from the corresponding author on reasonable request.
