# Supplementary material for: Neurotoxicity of organophosphate pesticides could reduce the ability of fish to escape predation under low doses of exposure
Source: Sci Rep. 2019 Jul 19;9:10530. doi: 10.1038/s41598-019-46804-6 (PMC6642105; doi:10.1038/s41598-019-46804-6)
Supplement: Supplementary file 1 — Supplementary material [file 41598_2019_46804_MOESM1_ESM.docx]

**Neurotoxicity of organophosphate pesticides could reduce the ability of fish to escape predation under low dose of exposure**

**Natalia Sandoval-Herrera^1,*^, Freylan Mena^2^, Mario Espinoza^1,3^ and Adarli Romero^1^**

^1^Escuela de Biología, Universidad de Costa Rica, 11501–2060 San José, Costa Rica

^2^Central American Institute for Studies on Toxic Substances/Instituto Regional de Estudios en Sustancias Tóxicas (IRET), Universidad Nacional, Campus Omar Dengo, Heredia, Costa Rica

^3^Centro de Investigación en Ciencias del Mar y Limnología (CIMAR), Universidad de Costa Rica, 11501–2060, San José, Costa Rica

**Supplementary material**

#### **Fish maintenance**

Fish were kept in a 60 L tank with continuous aeration and filtering through a canister filter system. The water was conditioned with similar physicochemical parameters of the site of collection (Conductivity = 16 μS / cm, T = 25º and dissolved oxygen = 5.5 mg/ml). Fish health and water parameters were monitored once a day. Approximately 25% of the water volume was replaced every week to avoid accumulation of nitrates residues in the water. The fish were acclimatized to the laboratory conditions for two weeks before any test. They were fed twice a day with commercial food *ad libitum* and exposed to a photoperiod of 14:10 light-dark hours.

#### **Exposure to Ethoprophos**

The dose of exposure was determined through preliminary tests. The criteria used to select the dosing was the minimum concentration to cause 50% of cholinesterase inhibition, since this was the most sensitive biomarker to measure. We tested four different concentrations: 0.25, 0.125, 0.0625, and 0.01 mg / L. The highest concentration tested (0.25 mg / L) corresponded to the minimum concentration of ethoprophos reported to cause ChE inhibition in *A. aeneus* ^39^*.*

For the exposure phase, the fish were separate in individual tanks of 4L capacity. An aliquot of ethoprophos was taken using a glass pipette from a stock solution (11.37 mg/L) and added to each treated tank, to obtain the nominal concentration of 0.01 mg/L. Fish were exposed for 48h with permanent aeration and 14:10 light-dark hours photoperiod. A 3 ml sample was collected at the beginning and the end of the exposure period (48 h) to assure the actual aqueous concentrations of ethoprophos.

#### **Neurotoxicity: Cholinesterase activity**

Cholinesterase (ChE) activity was determined colorimetrically using the method proposed by Ellman (1961), adapted to microplate by Guihermino (1996). No distinction between AChE and BuChE was done and the activity measured was expressed as total ChE. The Ellman assay was carried out by exposing the samples to a reaction solution (1 mM Acetylthio Choline and 0.1 mM DTNB). Absorbance was measured at 412 nm (ε = 13.6 mM^-1^cm^-1^) in a microplate reader (Multi Skan MS, type 352, Serial 35200, Labsystems, Helsinki, Finland) after 5 and 10 min the reaction was initiated. ChE activity was expressed as U mg, where U = nmol*min.

***Detoxification enzymes-*** Glutathione S-transferase (GST) conjugation activity was measured in liver homogenates using 1-chloro-2, 4-dinitrobenzene (CDNB) following the method of Habig et al., (1976). The reaction mixture contained 200 mM phosphate buffer (pH 6.5), 1 mM CDNB and 1 mM GSH. The formation of S-(2, 4-dinitrophenyl)-glutathione conjugate was evaluated by monitoring the increase in absorbance at 340 nm during 3 min (ε = 9.6 mM^-1^cm^-1^) and expressed as nmol/min/mg protein.

***Antioxidant enzymes-***Catalase (CAT) activity was measured in liver homogenates by the decrease in absorbance at 240 nm due to H_2_O_2_ consumption (ε = 40 M-1cm-1) according to Aebi et al (1974) and expressed as µmol/min/mg protein. The reaction volume and reaction time were 0.5 mL and 20 seconds respectively and contained 50 mM phosphate buffer, pH 6.5, 50 mM H_2_O_2_ (Ni et al., 1990).

***Oxidative stress-*** Lipid peroxidation (LPO) was measured by the thiobarbituric reactive species (TBARS) assay, which measures the production of malonaldehyde (MDA) that reacts with thiobarbituric acid (Oakes and Van Der Kraak, 2003). Measurement of TBARS was carried out following the method of Ohkawa et al (1979), using a Genesys 20, Thermo spectrophotometer. Briefly, 1 ml of 12% trichloroacetic acid, 0.8 ml of 60 mMTris–HCl pH 7.4 with diethylene triamine pentaacetic acid (DTPA) 0.1 mM and 1 ml of 0.73% thiobarbituric acid were added to 0.2 ml of liver homogenate. After a 60 min incubation in a bath at 100 °C, the solution was centrifuged at 2000 x g during 5 min and LPO levels were determined at 535 nm (ε = 156 mM-1cm-1) and expressed as nmol TBARS per mg of protein.

#### **Chemical determinations**

Chemical analysis of water samples was conducted at the Laboratorio de residues de plaguicidas (LAREP) of the Universidad Nacional, Costa Rica. Water samples from exposure tanks were analyzed using liquid chromatography-tandem mass spectrometry (LC-MS/MS), monitoring transitions for ethoprophos. The real concentration quantified was 0,014 ± 0,001mg/L. ^2^

The environmental water sample, collected at the site where fish were captured, was extracted by solid phase extraction (SPE) and pesticides were analyzed by gas chromatography with mass detector (GC-MS) and by liquid chromatography with photodiode array detector (LC-PDA).No trace of ethoprophos were detected, however residues of the insecticide diazinon were found. The concentration found was 0,06 µg/L, similar to levels reported for other sites in the region with relatively low impact by agricultural activities ^90^.
